# Versatile Functions of Somatostatin and Somatostatin Receptors in the Gastrointestinal System

**DOI:** 10.3389/fendo.2021.652363

**Published:** 2021-03-16

**Authors:** Bilal Haider Shamsi, Mahanand Chatoo, Xiao Kang Xu, Xun Xu, Xue Qun Chen

**Affiliations:** ^1^ Department of Neurobiology, Department of Neurology of the Second Affiliated Hospital, School of Brain Science and Brain Medicine, Hangzhou, China; ^2^ National Health Commission (NHC) and Chinese Academy of Medical Sciences (CAMS) Key Laboratory of Medical Neurobiology, Ministry of Education (MOE), Frontier Science Center for Brain Research and Brain Machine Integration, School of Brain Science and Brain Medicine, Zhejiang University, Hangzhou, China; ^3^ College of Renji, Wenzhou Medical University, Wenzhou, China

**Keywords:** somatostatin, somatostatin receptor, inflammation response, enteric nervous system, gastrointestine (GI)

## Abstract

Somatostatin (SST) and somatostatin receptors (SSTRs) play an important role in the brain and gastrointestinal (GI) system. SST is produced in various organs and cells, and the inhibitory function of somatostatin-containing cells is involved in a range of physiological functions and pathological modifications. The GI system is the largest endocrine organ for digestion and absorption, SST-endocrine cells and neurons in the GI system are a critical effecter to maintain homeostasis *via* SSTRs 1-5 and co-receptors, while SST-SSTRs are involved in chemo-sensory, mucus, and hormone secretion, motility, inflammation response, itch, and pain *via* the autocrine, paracrine, endocrine, and exoendocrine pathways. It is also a power inhibitor for tumor cell proliferation, severe inflammation, and post-operation complications, and is a first-line anti-cancer drug in clinical practice. This mini review focuses on the current function of producing SST endocrine cells and local neurons SST-SSTRs in the GI system, discusses new development prognostic markers, phosphate-specific antibodies, and molecular imaging emerging in diagnostics and therapy, and summarizes the mechanism of the SST family in basic research and clinical practice. Understanding of endocrines and neuroendocrines in SST-SSTRs in GI will provide an insight into advanced medicine in basic and clinical research.

## Introduction

Somatostatin (SST-14,28) is considered a universal endocrine molecule and a peptide hormone in the central nervous system (CNS), peripheral nervous system (PNS), and enteric nervous system (ENS) (1–5). It inhibits *via* different subtypes of SST receptors, and belongs to the superfamily of G protein-coupled receptors (GPCR) with 7-transmembrane domains. There are five SST receptors (SSTR1-5 and two isoforms SSTR2A, 2B), which are broadly expressed in the brain, spinal cord, dorsal root ganglion (DRGs), and ENS (1–6). The gastrointestinal (GI) system is recognized to be the largest endocrine organ for digestion and absorption by exocrine, endocrine, paracrine, and autocrine secretory effects in animals during GI physiological and pathophysiological processes. There are various SST-endocrine-cells embedded in the GI tract, which release gastrointestinal hormones to regulate GI function, such as SST producing-D cells from the stomach, intestine, and pancreas. SST in the GI system is involved in the inhibition of secretory activity and intestinal motility, blood flow, inflammation response, conduction of pain and sensation, and modulation of the release of hormone factors and other neurotransmitters, while SST-SSTRs mediate the release of gastric juice, intestinal juice, gastric acid, and other hormones *via* other endocrine factors (7–9). In addition, SST-SSTRs in non-GI tracts also are involved in digestion and absorption functions, such as the pancreas, liver, and gallbladder (10–12). The pancreas, containing SST producing-D cells, secretes the larger number of digestive liquids with digestive enzymes to mediate digestion and the absorption process. SST-SSTRs play an important role in the GI system *via* the neuroendocrine system. SST release from the GI is controlled by the vagus nerve and various local ENS neurotransmitters (13). SST-SSTRs of the brain and pituitary also impact on GI function *via* SST-SSTRs in the brain-gut axis and circulatory blood (14, 15). Advanced agonists and antagonists of SST-SSTRs are most commonly used in basic research and clinical practice (6, 16, 17), but the underlying mechanism is not fully understood. This mini-review will focus on the versatile functions of SST-SSTRs in the gastrointestinal system, and specifically in current advanced medicine.

## SST and SSTRs in Gastrointestinal System

SST and SSTRs are widely distributed in the GI system of the GI tract and non-GI tract (**Table 1**), while major endocrine cells (Enteric endocrine cells, EEC) and D cells in the stomach, intestine, and pancreas produce SST (7, 10). 90% of SST cells in the GI tract are endocrine cells, while 10% are neurons in the ENS (5). SST-SSTRs have an inhibition effect on the physiological functions of digestion and absorption in the GI system (8, 9). SST release from the pituitary and SST in circulatory blood could modulate GI function *via* its receptors.

**Table 1:** Distribution of SST and SST receptors in the gastrointestinal system.

Organ	Cell/Tissue	Humoral	SST	SSTR	References
Salivary gland				**+**	(18)
Oesophagus	S.P and M.P	Saliva	**+ + -**	**+ - -**	(5, 19)
Stomach	S.P D cells	Gastric juice	**+ + +** **+**	**+ + -** **+**	(5, 8, 9, 20–22)
Small intestine	S.P M.P EEC	Intestine juice	**+ + -** **+** **+**	**+ + -** **+** **+**	(5, 20)
Large intestine	S.P M.P D cells	**-**	**+ +** **+** **+**	**+ +** **+** **+**	(5, 20, 22)
Liver	Gallbladder	**-**	**+ - -**	**+ +**	(11, 12, 23)
Pancreas	D cells Cilia	Pancreatic juice	**+ + +** **+**	**+ + -** **+**	(24, 25)
Brain (Pituitary)	somatotropin cell	**-**	**+ +**	**+ +**	(26–28)
Blood vessels	smooth muscle cell	Circulatory blood	**+ + +**	**+ + -**	(15, 29)
Spinal dorsal horn (R4)	DRG	**-**	**+ +**	**+ +**	(18, 30–32)

S.P, submucosal plexus; M.P, myenteric plexus; + expression; - not reported; EEC, enteroendocrine cell; DRG, dorsal root ganglion.

## SST-Enteric Endocrine Cells and Nervous System in the Gastrointestinal tract

In the stomach, there are parietal cells, chief cells, neck mucous cells, G cells (Gastrin), delta (D) cells (Somatostatin), X cells (Ghrelin), and enterochromaffin-like cells (ECL, Histamine) for chemical digestion. SST is secreted mainly by delta cells of gastric mucosa. Closed-type SST-D-cells in corpus inhibit parietal cells, ghrelin cells, and ECL cells, and open-type SST-D-cells in antrum inhibit gastrin (G) cells and chief cells *via* paracrine inhibition. In SST transgenic mice, SST secretion from SST-D-cells is regulated by hormones, neurotransmitters, neuropeptides, and metabolites (17). In SST-cre mice, many signaling receptors of peptide YY (PYY), gastric inhibitory polypeptide receptor, cholinergic receptor muscarinic 4, calcitonin receptor-like receptor, trace amine receptor 1, and calcium-sensing receptor (Casr) are identified and highly enriched in SST-D-cells in the gastric epithelium by transcriptomic analysis (17, 33). SSTR2 is expressed on endocrine cells and intramural, myenteric, and mucosal nerve fibers, while SSTR1 and 3 are mainly distributed on smooth muscles and neuronal cells of submucosal and myenteric ganglia; these ganglia also contain SST^+^ neurons (5). 5-hydroxytryptamine (5-HT) is a marker for EEC of the GI mucosa; small proportions of 5-HT cells also containing gastrin or SST can be found in the stomach, and glucagon-like peptide (GLP-1) or SST in the large intestine (34). SST-positive **(**SST^+^) neurons in the ENS are changed in pig diabetes model (Streptozotocin); SST^+^ neurons increased only in the submucosal plexus in corpus of the stomach, and in the myenteric and submucosal plexus in all segments of the small intestine, but SST^+^ neurons decreased in the descending colon (20). In female pig stomach, SST^+^ immunoreactive neurons in the prepyloric area were increased in gastritis model (Aspirin, orally, 100mg/kg), in hyperacidity model (0.25MHCl intragastrical, 5ml/kg), and partial stomach resection model (21). Deletion of SST in multiple endocrine neoplasia type 1 (MEN-1) null mice caused hypergastrinemia and gastric carcinoids (35). After 12 ws-15% fructose in drinking, SST-D-cells were diminished in gastric corpus and increased in the antrum in male-Wistar rats (36). C-X-C motif chemokine ligand-14 (CXCL14) immunostaining endocrine cells and SST^+^ cell and nerve fibers co-expressed at the lamina propria and submucosal and muscular layer from the stomach to rectum in mice (37). This indicated that the mechanism of SST might involve immune function and inflammation response and crosstalk. There is localization of muscarinic acetylcholine receptors (M4) on D-cells in the mice stomach, and serum SST levels in M4-KO-mice were higher than WT-mice; the activation of M4-receptors inhibits SST release from D-cells and minimizes SST inhibition for gastric acid release through SSTR2, which in turn enhances acid response by M3-receptors on parietal cells (38). In pig stomach tissues, the study demonstrated amino acids can increase gastrin and SST secretion, and the Casr-dependent pathway modulated H^+^-K^+^-ATPase activity (39).

In the small intestine, SST-D-cells are located in the lamina propria and between the epithelial cells of the crypts, while the SST^+^-neurons are located in the submucosal and myenteric plexus and innervate intestinal smooth muscles, submucosal layer, and the mucosa. SST^+^ neurons are type-II-Dogiel cells with branched dendrites and one long axon (5, 7). EEC expressed urotensin 2B in the jejunum and colon; these cells inhibit GLP-1 secretion through SSTR5 in a paracrine manner (40, 41). Occasionally SST-cell will colocalize with 5-HT in mice small intestine (34). The transcription factor “aristaless-related homeobox” (Arx) is specifically expressed in intestinal endocrine cells. In neonatal Arx-KO mice, many intestinal hormones, such as cholecystokinin, secretin, neurotensin, glucose dependent insulinotropic peptide, GLP-1, and GLP-2, did not express in GI, but SST and chromogranin A (CGA) were significantly upregulated in the duodenum. Also there is loss of lipid transport in duodenal enterocytes, more lysozyme-positive Paneth cells, and increased-antimicrobial peptides in the steatorrhea mice (42). In mouse constipation model (Carbon bioactivated), Lactobacillus fermentum CQPC03 (LF-CQPC03) relieved constipation symptoms in mice, and serum levels of SST were lower, and levels of gastrin, endothelin, and acetylcholinesterase were higher in mice after CQPC03 treatment (43). In arthritic-rats (Curcumin,100mg/kg, oral), SST secretion elevated from the endocrine cells in the small intestine, and SST exhibited an anti-arthritic effect *via* cAMP/PKA and Ca^2+^/CaMKII mechanism. SST depletor (CSH) and non-selective SST receptor antagonist (C-SOM) abolished the inhibitory effect of curcumin on arthritis (44), this supported the hypothesis that SST from GI mediates the inflammation response. In hereditary transthyretin amyloidosis patients undergoing evaluation for liver transplantation, SST (Octreotide) administration induced fasting motility with more daytime phase III migrating motor complexes, and higher frequency of low-amplitude complexes in 24h duodenojejunal manometry; there was a delay in SST (Octreotide) response in late-onset patients (50 years of age) (45).

In guinea pig large intestine, round or oval SST-neurons in the submucous plexus have oral projections, and in myenteric plexus both have oral and caudal projections. SST-neurons in the proximal colon are more abundant in the submucosal plexus as compared to the myenteric plexus, and in the distal colon myenteric plexus SST-neurons are denser and have visible varicosities. Two types of SST-neurons have been identified in pig: type-V descending interneurons located in the myenteric and outer submucosal plexus and type-IV neurons secretomotor neurons in all types of intramural plexuses (5). In the colon, there was a lower number of epithelium SST-cells in colitis (Dextran-sulfate-sodium-induced) rat model and higher epithelium SST-cells in colitis (Trinitrobenzene sulfonic acid) rat model (46, 47). In colitic-mice, SST (Octreotide) stimulated colonic sodium/hydrogen exchanger 8 (NHE8) expression, while SSTR2 agonist (Seglitide) and 5 agonist (L-817/818) restored NHE8 expression *via* by suppressing ERK1/2 phosphorylation (48). SST exposure (LS174T-cells) stimulated colonic MUC2-mucin2 expression and mucus secretion in human goblet-like cells, this was blocked by SSTR5 siRNA transfection and SSTR5 antagonist (L817/818) (49). Colon tissue from selenoprotein glutathione peroxidase 2 (GPx2) knockout mice, under selenium deficiency or adequate Se supplementation, showed a downregulation of SST mRNA expression (50). GPx2 might be important for intestinal epithelium function. By single cell transcriptomic profiling from the colon, seven EEC subgroups were identified, four clusters expressed high levels of Tph1 (ECM-cells, 50%), two clusters were enriched for proglucagon (GCG) and PYY (L-cells, 40%), and the last cluster expressed high levels of SST, which is characteristic of D-cells (10%) (51). After change in dietary habits in irritable bowel syndrome (IBS) patients, there was an increase in SST-cells in the rectum epithelium and symptomatic relief (52, 53). Oral treatment (Lactobacillus plantarum ys2) in carbon-induced constipation Kunming mice reduced serum SST level, promoted gastrointestinal peristalsis, and reduced the first black stool defecation time (54). SST immunoreactive neurons co-expressed with P2X1-receptor (ATP receptor) was detected in mouse myenteric and submucosal plexuses (55). Using intrinsic markers targeting vagal and spinal sensory, sympathetic, and parasympathetic axons, the spatiotemporal map showed extrinsic axons project along the myenteric plexus and keep intimate contact with enteric neurons in whole gut in mice E9.5-E16.5 (56). This proposed that SST-neurons can crosstalk with other neurotransmitters in the parasympathetic and sympathetic system and ENS of the gastrointestinal tract.

## SST-Endocrine Cells in Non-Gastrointestinal Tract

Somatotropic cells in the pituitary control GI function *via* brain-gut axis and circulation. Classical SST and growth hormone-releasing-hormone receptor (GHRH) in the hypothalamus affects negative and/or positive control growth hormone (GH) transcription and release in mammals. Currently, the structural and functional connection between Pomc-neurons and the somatotropic hypothalamo-pituitary axis have been reported in larval zebrafish. Excessive feeding induced leptin resistance and decreased-Pomc expression; Pomc-neurons stimulating SST-neurons result in reduced-growth hormone. So, SST-neurons mediate faster somatic growth, this suggests that a Pomc-SST-GH axis might be involved in metabolism and homeostasis (57). Both SSTR2 and SSTR4 in the hippocampus selectively inhibit HPA axis activation induced by stress but regulate anti-depressive and anti-anxiety effects through different mechanisms in rats (26). SSTR5 modifies HPA axis stress response and attenuates corticotroph responses to corticotropin releasing factor (CRF) by post-transcriptionally suppressing CRFR1 expression and function *via* miR-449 (27). In pituitary AtT20-cells, CRF (100nM) induced rapid Rab10-dependent resurfacing of endocytosed SSTR2 *via* CRFR1, providing a feedback mechanism to inhibit CRFR signaling (28). Hypoxia stimulated expression of SSTmRNA and protein in the periventricular nucleus of the hypothalamus and decreased GH release from pituitary and body weight gain in rat. CRFR1 and SST were involved in downregulated-mRNA of pituitary GH and upregulated-mRNA of hepatic insulin-like growth factor-I (58–61). The activation of central SST signaling induced a robust stimulation of food and water intake *via* SSTR2. Brain SST contributes to the orexigenic and dipsogenic responses during the dark-phase in rodents (62). SST released from the capsaicin-sensitive sensory nerves mediates analgesic and anti-inflammatory effects *via* the SSTR4-receptor, while orally novel SSTR4 agonists exert a potent anti-hyperalgesic effect in chronic neuropathy mice model (30).

In addition, SST, secreted by pancreatic D-cells (δ-cells), is a powerful paracrine inhibitor for insulin and glucagon secretion (from α-cells and β-cells). D-cells comprise only ~5% of the cells of the pancreatic islets. Some factors (Insulin, Glucagon, Urocortin 3, and GABA) released by neighboring α-cells or β-cells amplify the glucose-induced effects on SST secretion from D-cells, and SST acts locally within the islets as a paracrine or autocrine inhibitor of insulin (10). Glucose stimulates SST secretion in δ-cells *via* cAMP-dependent [Ca^2+^]^i^ release (63). SST-D-cells contain ATP-sensitive K^+^ channels which close at high glucose levels; this closure channels initiate membrane depolarization and increase SST secretion (63). Meanwhile, stimulation of SST secretion also depends on sodium/glucose cotransporter 2 (SGLT2), by which insulin can inhibit glucagon release by an indirect paracrine mechanism (64). After the ablation of insulin-secreting β-cells in mice, some glucagon-producing α-cells and SST-producing D-cells become insulin-expressing cells (65). α-cells can reprogram to produce insulin from puberty through to adulthood to aged individuals, even a long time after β-cell loss in mice, whereas only juvenile’s SST-producing-D-cells convert to insulin producer by dedifferentiation, proliferation, and re-expression of developmental regulators [FoxO1 (Forkhead box protein O1) and downstream effectors] (24). The multiple intra-islet cell interconversion mechanisms offer new insight for future clinical therapy. Reduced SST secretion in isolated islets induced hypersecretion of glucagon in high fat diet-fed female mice, however, this cannot be suppressed by exogenous SST (SST-14, 1.34mL/min/mg pancreas) (66). Cilia in pancreatic tissues are hubs for cellular signaling and are involved in proper development of pancreatic epithelium and β-cell morphogenesis *via* SSTR3. A paracrine negative feedback role for β-cell ciliary SSTR3 regulates insulin secretion. Immunohistochemical and electron microscopic study found abundant SSTR3-expressing solitary cilia in insulin- and GH-secreting cells in mouse. SSTR3 was restricted to the plasma membrane of cilia, but not at the cell body. The primary cilia in the islet-cells were longer than those in the pituitary cells and extended for a long distance in the intercellular canaliculis endowed with microvilli (25). The study demonstrated the mechanisms of tight glycemic control in islet-D-cells, mice urocortin-3 co-released with insulin, and increased glucose-stimulated SST secretion *via* cognate receptors, this indicated SST-dependent negative feedback control of insulin secretion (67).

## Mechanisms of SST–SSTRs in Endocrine Cells

SST is a cyclic hormone-release inhibitory peptide with 2-3 minutes half-life that has high binding affinity to all of its five SST-receptors (SSTRs) in the GPCR superfamily. SST negatively regulates the release of multiple hormones and cell proliferation *via* activation of its cognate receptors. The five subtypes of SSTR1-SSTR5 are coupled with inhibitory G protein Gi/o (**Table 2**) and are involved in motility, mucous and hormone secretion, blood vessels contractility, inflammation responses, and microbiotal flora (9, 54). Phosphorylation and dephosphorylation of SSTRs at C-terminal or serine and/or threonine residues is involved in fine‐tuning signalling (75). A novel higher selective monoclonal antibody for extracellular domain of SSTR2 binds the surface of neuroendocrine tumor (NET) cells *via* signalling cascades and reduce tumor growth. Phosphosite-specific antibodies for human SSTR2 and SSTR5 monitor the spatial and temporal dynamics of SSTRs’ phosphorylation and dephosphorylation (75, 80). Based on the integrated physiological regulation mechanism of the neuroendocrine system, a high combination of chimeric molecules for NET and cancer have emerged (73, 81) in 2D- and 3D-cultures. SST-D2R multi-receptor targeting drugs inhibit CgA and serotonin secretion, but not NET cell growth (81). Overexpressed-SSTR2 in pancreatic human NETs demonstrated that fluorescence of SSTR2 receptor-mediated uptake was observed at the macro-, meso-, and microscopic scales, thus displaying specific SSTR2-digital image pathological findings, such as tumor boundaries and location (82).

**Table 2:** Effect of SST and SSTRs in the gastrointestinal system for basic research and clinical medicine.

Function		Gastrointestinal system				Neuroendocrine and endocrine tissue	References
Name		Secretion	Movement	Blood vessel	Inflammation	Proliferation (Tumor/cancer)	
SST (analogues)	Octreotide (Sandostatin) (Vapreotide, Seglitide)	↓ (gastric juice, gastric acid)	↓ (gastric emptying, segmental motility)	↓	↓	↓ normal tissue and tumor, Severe diarrhea, Carcinoid syndrome with unresectable, metastasized tumor ↓ normal tissue and tumor ↓ normal tissue and tumor For Imaging in nuclear medicine	(11, 16, 29, 68–72)
	Lanreotide	↓	↓ mass peristalsis	↓	↓		
	Pasireotide Cortistatin	↓	↓ defecation reflex	↓	↓ ↓↓		(16, 19, 29, 70, 73, 74)
	Somatoprim (heptapeptide) In-111-Pentetreotide	↓	↓	↓	↓		(16, 75) (76)
GPCR-SST receptor (agonist or antagonist)	R1(Gαi3, 1/2)	↓	↓	―	―		(6)
	R2(Gαi3)	↓	↓	―	―		(6)
	R3(Gα1i)	↓	↓	―	―		(6)
	R4(Gi)	↓	↓	―	―		(6)
	R5(Gi/Gq)	↓↓	↓↓	↓↓	―		(6)
SST receptor phosphorylation or dephosphorylation (agonist)	C-terminal serine/threonine			↓	↓↓	↓↓	(6, 75, 77)
Chimeric molecule, Combination (+agonist or antagonist)	+DA receptor 2 (D2DR, Dopastatin)	↓	↓	―	―	↓non-hormone producing tumor	(75)
	+ GH receptor (pegvisomant)	↓	―	―	―	↓ solid tumor or cancer	(69–71, 78)
	+mTOR inhibitor (rapamycin,everolimus)	↓	―	↓↓	↓	↓ solid cancer	(29, 71, 79)
Antibody for human SST 2/5 receptors						Predictive for diagnostic and intervention	(80)


Combination (+chem-molecules)	+α interferon (tamoxifen)	―	―	↓	↓	↓ cancer in GI and others	(11, 78)
	+anti-VEGF (bevacizumab)	―	―	↓↓	↓		(22, 72, 79)

GPCR, G protein-coupled receptor; R1-R5, receptor 1-5; DA, Dopamine; D2DR, D2 of dopamine receptor; VEGF, Vascular endothlial growth factor; Gα,i,q, subtype of G proteins; mTOR, Mammalian target of rapamycin; GH, Growth hormone; GI, Gastrointestinal tract; ↓ = Downregulation; ― = Unchanged.

## SST-SSTRs in Basic Research and Clinical Medicine

SST analogues (Octreotide, Lanreotide, and Pasireotide (for SSTR2,5)) are widely used as first-line treatment for perioperative period, metabolic diseases, and tumor control (68, 78, 83). Octreotide, lanreotide, and pasireotide are applied to acromegaly, Cushing’s syndrome, and carcinoid syndrome, respectively (69, 70, 74). SST (Pasireotide 10-30mg/4ws/year) decreased urinary free cortisol and late-night salivary cortisol to normal levels in patients with Cushing’s disease (19). NETs are heterogeneous malignancies in different neuroendocrine systems and higher incidents in the GI and Non-GI tract of SST analogs have been confirmed in antisecretory and antitumor efficacy (19, 68–70, 74, 78, 79, 83). The FDA approved peptide-receptor radionuclide therapy (Lutathera^®^) for gastroenteropancreatic NETs; the treatment can improve patient survival (71, 79). This mini review presents the current drugs used in basic and clinical practice (**Table 2**). Anti-SSTR2 antibody drugs have been developed for NET diagnosis and therapy (79, 80). Small molecule SSTR4 agonists (4-phenetylamino-7H-pyrrolo[2,3-d] pyrimidine derivatives, 100µg/kg, oral) inhibited neurogenic inflammation and neuropathic hyperalgesia (Sciatic nerve-ligation model) in rats (31). Escalated doses of SST analogs (Octreotide, >30mg or Lanreotide, >120mg/d, 28days) were well tolerated with antiproliferative effects in neuroendocrine neoplasms (NEN) patients (72). Recently, phosphate-specific antibodies have shown agonist-selective properties for individual tumor tissues (75). NET patients overexpressed SSTR2 at high density; the membrane expression of SSTR2 in tumors cells is ~20-fold higher than that of normal cells (79, 84). Immunohistochemistry analysis showed over 70% of NET patients with expressed-SSTR2 (79, 84). SSTR expression is a biomarker of NEN biology and immunohistochemical staining SST, while SSTR and CGA are candidates for prognostic information and risk-stratification in clinic. SSTR2a was a positive prognostic marker for pancreatic NEN (79, 85). Monitoring of treatment of SST analogues and changed-circulating CGA levels can predict disease recurrence, outcome, and efficacy (29). It is very interesting that the image for SST-SSTRs showed nonspecific accumulation in activated immunological cells, lymphocytes, epithelioid cells, monocytes, and blood vessels (76). Internalization of the radiolabeled agonist was mandatory for SSTR-mediated imaging and therapy, but radiolabeled SSTR antagonists might perform better in preclinical work (86). SST (0.25mg, and prophylactical SST long-term (0.25mg/h for 10h iv) decreased post-endoscopic retrograde cholangiopancreatography (ERCP) pancreatitis and post-ERCP hyperamylasemia (87). However, current effective preventive strategy suggested rectal non-steroidal anti-inflammatory drugs and pancreatic stent placement (88), and a meta-analysis showed SST lacks the benefit in patients with ERCP after short-term infusion, but gains an advantage in single-bolus or long-term injection (89). Therefore, the risk *vs* benefit of SST and analogues should be carefully assessed in patients (88). Long-acting SST analogues (Octreotide, 20mg/m or Lanreotide, 90mg/28 d, 12months, im) decreased blood transfusion in patients with refractory bleeding from gastrointestinal angiodysplasias (90). SST prolongs progression-free survival (PFS) in individual precision medicine; global multicenter studies have confirmed high dose-SST (Octreotide, 30mg/4ws) (Lanreotide, 120mg/4ws) is an active and safe option in patients with progressive well-differentiated gastroenteropancreatic NETs, and an independent individualized prediction model could be a valuable tool for making treatment decisions in clinical practice for SST-treated patients (91–93). Therefore, powerful prediction tools, drug-combination, and conjugated-medicine are important to limit the side effects of SST analogues in fatigue, diarrhoea, constipation, abdominal pain, nausea, cholelithiasis and hyperinsulinism, and necrotizing enterocolitis in infants (72, 94, 95). SST analogues might disturb infant development and neuroendocrine system.

## Conclusion

SST-endocrine cells and neurons are a critical effector to maintain homeostasis *via* SSTRs 1-5 and co-receptors. SST-SSTRs are involved in chemo-sensory, secretion, motility, and inflammation responses by autocrine, paracrine, endocrine, and exoendocrine pathways. SST is also a powerful inhibitor for tumor cell proliferation, severe inflammation, and perioperation, and is listed as a first-line anti-cancer drug in clinical practice. New development prognostic SS-SSTRs-markers, phosphate-specific antibodies, and molecular imaging have emerged in diagnostics and therapy. Dis-inhibition and network of SST-SSTRs in the PNS of the sympathetic and parasympathetic nerve system and special DRG should be explored. Understanding the mechanisms of neuroendocrine in SST-SSTRs in the GI will provide an insight into advanced medicine.

## Author Contributions

BHS, MC, and XQC drafted the manuscript. XQC supervised the project and conceived of the study. XKX contributed to discussion and manuscript editing. XX contributed to **Table 2**. All authors contributed to the article and approved the submitted version.

## Funding

This research was supported by the National Natural Science Foundation of China (grant number 81930054, grant number 81741120) and the Ministry of Science and Technology of China, National Basic Research Program (973) of China (grant number 2012CB518200).

## Conflict of Interest

The authors declare that the research was conducted in the absence of any commercial or financial relationships that could be construed as a potential conflict of interest.
